# Supplementary material for: The mechanism for directional hearing in fish
Source: Nature. 2024 Jun 19;631(8019):118–24. doi: 10.1038/s41586-024-07507-9 (PMC11222163; doi:10.1038/s41586-024-07507-9)
Supplement: Supplementary file 1 — Supplementary Tables 1 and 2 and References. [file 41586_2024_7507_MOESM1_ESM.pdf]

nature portfolio

https://doi.org/10.1038/s41586-024-07507-9

Supplementary information

The mechanism for directional hearing in fish

In the format provided by the
authors and unedited

Supplementary Table 1 (caption on the next page)

Study type	Species	WA	Plane	Dominant frequency (red: f < 20 Hz, blue ≥ 200, purple in-between)	Reverberations	P measured	M measured	LL ablation	Other cues excluded (timbre/ vision)	Klinotaxis excluded	Resolution of 180° ambiguity	Number of individuals tested (red: n < 10)	Important cues (noted by authors)	Reference
I	Goldfish *(Carassius auratus)*	YES	h	100, 150, 1500 Hz	HIGH	YES	NO	NO	NO	YES	YES	2-4 (across 7 conditions)	-	Moulton, Dixon[24] 1967
V	Atlanto-Scandian herring	NO	h	20 Hz - 6 kHz	LOW	YES	NO	NO	YES	NO	YES	Unclear	-	Olsen[20] 1969
II	Ballan wrasse *(Labrus bergylta)*	NO	h	115 Hz	LOW	YES	YES	NO	NO	YES	NO	1	-	Schuijf, Baretta, Wildschut[81] 1972
IV	Hawaiian squirrelfishes *(Myripristis berndti, M. argyromus)*	NO	h,v	< 1.2kHz	LOW	YES	NO	NO	YES	NO	YES	Tested in groups	(P),M	Popper, Salmon, Parvulescu[82] 1973
II	Haddock, Atlantic cod *(M. aeglefinus, Gadus morhua)*	NO	h	Cod: 40- 200 Hz Haddock: 50- 380 Hz	LOW	YES	NO	NO	NO	YES	NO	2 haddock, 3 cod	-	Chapman[11] 1973
II	Haddock, Atlantic Cod *(M. aeglefinus, Gadus morhua)*	NO	h	60 - 380 Hz	LOW	YES	NO	NO	NO	YES	YES	3 haddock, 9 cod	-	Chapman, Johnstone[12] 1974
III	Atlantic cod *(Gadus morhua)*	NO	h	75 Hz	LOW	YES	YES	NO	NO	YES	YES	2	P, M	Schuijf[7] 1975
III	Atlantic cod *(Gadus morhua)*	NO	h	75 Hz	LOW	YES	YES	NO	NO	YES	YES	2	P, M	Schuijf, Buwalda[8] 1975
III	Ide *(Leuciscus idus)*	YES	h	75 Hz	LOW	YES	NO	NO	NO	YES	YES	2	-	Schuijf, Visser, Willers, Buwalda[83] 1977
II	Atlantic cod *(Gadus morhua)*	NO	v	110 Hz	LOW	YES	NO	NO	NO	YES	NO	3	-	Hawkins, Sand[13] 1977
I	Herring *(Clupea harengus)*	NO	h	61 - 160 Hz	HIGH	YES (partially)	YES (partially)	NO	YES	YES	YES	Tested in groups	-	Blaxter, Gray, Denton[21] 1981
I	Goldfish *(Carassius auratus)*	YES	h	Dropping ball next to fish	HIGH	NO	NO	NO	NO	YES	YES	1	-	Eaton, Lavender, Wieland[25] 1981
IV	Plainfin midshipman *(Porichthys notatus)*	NO	h,v	95 Hz	HIGH	NO	NO	NO	YES	NO	YES	Unclear	-	Ibara, Penny, Ebeling, Dykhuizen, Cailliet[15] 1983
II	Atlantic cod *(Gadus morhua)*	NO	h,v	120 Hz	LOW	YES	YES	NO	YES	YES	YES	2	P, M	Buwalda, Schuijf, Hawkins[14] 1983
IV	Bicolor damselfish *(Pomacentrus partitus)*	NO	h	250-750 Hz	LOW	NO	NO	NO	YES	NO	YES	21	-	Myrberg, Mohler, Catala[84] 1986
I	Goldfish *(Carassius auratus)*	YES	h	Dropping ball next to fish	HIGH	NO	NO	NO	NO	YES	YES	7	-	Eaton, Emberley[26] 1991
I	Angelfish *(Pterophyllum eimekei)*	?	h	Slamming against tank	HIGH	NO	NO	NO	YES	YES	YES	15	-	Domenici, Blake[85] 1993
IV	Bicolor damselfish *(Pomacentrus partitus)*	NO	h,v	350-1000 Hz	LOW	YES (partially)	NO	NO	YES	NO	YES	9	-	Kenyon[86] 1994
I	Herring *(Clupea harengus)*	NO	h	100 Hz	HIGH	NO	NO	NO	YES	YES	YES	3	-	Domenici, Batty[22,23] 1994, 1997
I	Goldfish *(Carassius auratus)*	YES	h	Broadband, single push/pull	HIGH	YES	NO	YES	YES	YES	YES	> 16	P, M	Canfield, Rose[27] 1996
I	Cichlid *(Haplochromis burtoni)*	NO	h	Broadband, single push/pull	HIGH	YES	NO	NO	YES	YES	YES	> 16	-	Canfield, Rose[27] 1996 (same as above)
I	Goldfish *(Carassius auratus)*	YES	h	Pulse, (rise times 0.4 - 1.0 ms)	HIGH	YES	- (estim.)	NO	YES	YES	YES	5	M	Lewis, Rogers[28] 1998
IV	Plainfin midshipman *(Porichthys notatus)*	NO	h	80 - 140 Hz	HIGH	YES	NO	NO	YES	NO	YES	Unclear	-	McKibben, Bass[16] 1998
I	Goldfish *(Carassius auratus)*	YES	h	200 Hz	HIGH	YES (partially)	NO	NO	YES	YES	YES	51	-	Preuss, Faber[29] 2003
I	Roach *(Rutilus rutilus)*	YES	h	6.7 Hz swing system (infrasound)	HIGH	YES	YES	YES	YES	YES	YES	54 fish,tested in groups of 7-8	P, M	Karlsen, Piddington, Enger, Sand[87] 2004
IV	Plainfin midshipman *(Porichthys notatus)*	NO	h	90 Hz	HIGH	YES	YES	NO	YES	NO	YES	62	P, M	Zeddies, Fay, Alderks, Shaub, Sisneros[17] 2010
IV	Plainfin midshipman *(Porichthys notatus)*	NO	h	80 Hz, 90 Hz	HIGH	YES	YES	NO	YES	NO	YES	84	P, M	Zeddies, Fay, Gray, Alderks, Acob, Sisneros[18] 2012
I	Goldfish *(Carassius auratus)*	YES	h	200 Hz	HIGH	YES (partially)	NO	YES[1]	YES	YES	YES	> 30	LL	Mirjany, Preuss, Faber[30] 2011
IV	Plainfin midshipman *(Porichthys notatus)*	NO	h	75 Hz, 80 Hz	HIGH	YES	YES	YES	YES	NO	YES	108	P, (M)	Coffin, Zeddies, Fay, Brown, …, Sisneros[19] 2014
I	*Danionella cerebrum*	YES	h	Pulse, 780 Hz, 0.66 ms rise time	Actively canceled	YES	YES	YES	YES	YES	YES	139	P, M	This study

Supplementary Table 1 | Literature reporting directional hearing behavior in teleosts with swimbladder: Literature was sorted chronologically and categorized into five study types: I) Startle II) Discrimination task III) Conditioned phonotaxis IV) Innate phonotaxis V) Sound avoidance. We indicate whether the tested species has a Weberian apparatus (WA), whether directional hearing was tested in the horizontal (h) or in the vertical plane (v), whether the experiment was performed in an environment with fewer reverberations (e.g. open water), whether pressure (P) and particle motion (M) were measured, whether the lateral line (LL) was necessary for directional hearing behavior, whether other cues such as small variation in sound timbre or visual cues could be excluded, whether the step-by-step sampling of an amplitude gradient (klinotaxis) can be ruled out, and whether the 180° ambiguity was resolved (either by localization relative to sound or discrimination of opposite sources). Early studies were predominantly performed in the free field in low reverb environments and involved conditioning paradigms. Due to the complexity of these experiments, they were only performed on a few fish. Since the 1980s, experiments were mostly performed in reverberation-rich lab settings that necessitated the measurement of pressure and particle motion. Reverberations were classified as low in open water conditions, and high otherwise. Footnote 1: Myrjani et al. 2011 found no evidence for directional hearing after ablating the lateral line.

Sound configuration (+/- refers to pressure polarity)	Idealized speaker signal (sign)	Factor α_i for bound to least square solver	Actual speaker signals, targeted for center (peak absolute amplitude)	Target (p,a_x,a_y)	Sound targeting, correlation with target (p,a_x,a_y)
Single left speaker +	$[+,0]_h$ $[0,0]_o$	$[1,0]_h$ $[0.1,0.1]_o$	[**1.19**,0.00]$_h$ [0.26,0.28]$_o$	(+,+,0)	(0.98,0.98,x)
Single left speaker -	$[-,0]_h$ $[0,0]_o$	$[1,0]_h$ $[0.1,0.1]_o$	[**1.19**,0.00]$_h$ [0.26,0.28]$_o$	(-,-,0)	(0.98,0.97,x)
Trick conf., left +	$[-,0]_h$ $[+,+]_o$	$[-,0]_h$ $[1,1]_o$	[**1.14**,0.00]$_h$ [**1.46,1.65**]$_o$	(+,+,0)	(0.98,0.97,x)
Trick conf., left -	$[+,0]_h$ $[-,-]_o$	$[-,0]_h$ $[1,1]_o$	[**1.14**,0.00]$_h$ [**1.46,1.65**]$_o$	(-,-,0)	(0.97,0.97,x)
Single right speaker +	$[0,+]_h$ $[0,0]_o$	$[0,1]_h$ $[0.1,0.1]_o$	[0.00,**1.74**]$_h$ [0.29,0.32]$_o$	(+,-,0)	(0.98,0.97,x)
Single right speaker -	$[0,-]_h$ $[0,0]_o$	$[0,1]_h$ $[0.1,0.1]_o$	[0.00,**1.74**]$_h$ [0.29,0.32]$_o$	(-,+,0)	(0.97,0.96,x)
Trick conf., right +	$[0,-]_h$ $[+,+]_o$	$[0,-]_h$ $[1,1]_o$	[0.00,**1.67**]$_h$ [**1.40,1.58**]$_o$	(+,-,0)	(0.97,0.97,x)
Trick conf., right -	$[0,+]_h$ $[-,-]_o$	$[0,-]_h$ $[1,1]_o$	[0.00,**1.67**]$_h$ [**1.40,1.58**]$_o$	(-,+,0)	(0.97,0.97,x)
Pressure conf. +	$[+,+]_h$ $[+,+]_o$	$[1,1]_h$ $[1,1]_o$	[**0.39,0.51**]$_h$ [**0.49,0.54**]$_o$	(+,0,0)	(0.98,x,x)
Pressure conf. -	$[-,-]_h$ $[-,-]_o$	$[1,1]_h$ $[1,1]_o$	[**0.39,0.51**]$_h$ [**0.49,0.54**]$_o$	(-,0,0)	(0.98,x,x)
Particle motion conf. (1)	$[+,-]_h$ $[0,0]_o$	$[1,1]_h$ $[1,1]_o$	[**0.72,0.66**]$_h$ [0.15,0.17]$_o$	(0,+,0)	(x,0.98,x)
Particle motion conf. (2)	$[-,+]_h$ $[0,0]_o$	$[1,1]_h$ $[1,1]_o$	[**0.72,0.66**]$_h$ [0.15,0.17]$_o$	(0,-,0)	(x,0.98,x)

Supplementary Table 2 | **Speaker signals:** The setup comprises four speakers to deliver target waveforms to the current fish position (values shown are for the center grid point position). We label both horizontal speakers $[a,b]_h$ and both orthogonal speakers $[c,d]_o$ to indicate whether they should be active with positive polarity [+], with negative polarity [-], or not at all [0] in an idealized scenario. In this notation, a positive waveform from left $[+,0]_h$ $[0,0]_o$ creates pressure and particle motion target signature (p=+, a_x=+, a_y=0) and an amplitude-inverted waveform from the left $[-,0]_h$ $[0,0]_o$ creates (-,-,0). In reference to Schuijf's model, the phase relationship between p and a_x is inverted if these sounds are played from right, with $[0,+]_h$ $[0,0]_o$ creating (+,-,0) and $[0,-]_h$ $[0,0]_o$ creating (-,+,0). We set the factor α_i that constraints the deconvolution in sound targeting separately for each speaker to the listed values. Thus constraint, the deconvolution in sound targeting creates speaker signals with the listed absolute amplitudes (in Volts, speakers are driven with 10x of this amplitude), example waveforms shown in Extended Data Fig. 3. Peak absolute amplitudes of waveforms at speakers after sound targeting are highest only for those speakers active in the idealized speaker signal column (bold). The target pressure and acceleration waveforms are realized at the target (≥ 0.96 correlation between measurements and target waveforms).

SI References

81. Schuijf, A., Baretta, J. W. & Wildschut, J. T. A field investigation on the discrimination of sound direction in Labrus berggylta (pisces: Perciformes). Neth. J. Zool. 22, 81–104 (1972).
82. Popper, A. N., Salmon, M. & Parvulescu, A. Sound localization by the hawaiian squirrelfishes, Myripristis berndti and M. argyromus. Anim. Behav. 21, 86–97 (1973).
83. Schuijf, A., Visser, C., Willers, A. F. M. & Buwalda, R. J. A. Acoustic localization in an ostariophysian fish. Experientia 33, 1062–1063 (1977).
84. Myrberg, A. A., Mohler, M. & Catala, J. D. Sound production by males of a coral reef fish (Pomacentrus partitus): its significance to females. Anim. Behav. 34, 913–923 (1986).
85. Domenici, P. & Blake, R. W. The effect of size on the kinematics and performance of angelfish (Pterophyllum eimekei) escape responses. Can. J. Zool. 71, 2319–2326 (1993).
86. Kenyon, T. N. The significance of sound interception to males of the bicolor damselfish,Pomacentrus partitus, during courtship. Environ. Biol. Fishes 40, 391–405 (1994).
87. Karlsen, H. E. Infrasound initiates directional fast-start escape responses in juvenile roach Rutilus rutilus. J. Exp. Biol. 207, 4185–4193 (2004).
